# Hip Joint Disease

**Published:** 1891-04

**Authors:** P. H. Fitzhugh


### "HIP JOINT DISEASE."

BY P. H. FITZHUGH, M. D.

SYNONYMS: Morbus Coxarius; Morbus Coxæ; Hip Disease; Turberculous Disease of the Hip; Chronic Epiphysitis of the Hip; Medello-Arthritis; Coxalgia; Coxitis; Chronic Articular Ostitis of the Hip; Coxo-tuberculose (Lannalongue).

Webster defines the hip as "the projecting part of the trunk of an animal formed by the lateral parts of the pelvis and the hip joint with the flesh covering them; the haunch."

Gould gives as the hip, "the upper part of the thigh at its junction with the buttocks."

Haunch is "that part of the body including the hips and buttocks."—(Gould.)

Webster gives as the definition of haunch "the hip, that part of the body of man and quadrupeds which lies between the last ribs and the thigh."

From the above definitions it will be seen that the term hip, is employed rather indefinitely by the profession as well as the laity, to include not only the immediate structures which enter into the formation of the joint proper, but also those both hard and soft which contribute to the function and protection of the same, even to the fascia and integument.

From this wide range confusion in properly classifying diseases of and about the joint arises.

For instance, when one makes the assertion that he has cured a case of hip-disease with restoration to perfect function, you will frequently learn on questioning that he can give no absolute anatomical grounds for his diagnosis. The fact is, that, so long as our definition of hip is this broad, just so long will the term hip-disease be misleading, for a scratch, a bruise, a boil, anywhere from about the upper quarter of the thigh to and including the pelvis is on the hip.

It is all important for the orthopedist to acquaint himself from personal observation of the contour of a normally formed hip, the appearance of the lines and dimples into which it is thrown, as the subject assumes different attitudes. I can do no better than quote from Dr. Gibney on this subject.

"The prominences of the nates of course stand more conspicuously as the erect position is assumed; the fullness or flabbiness indicating health or the reverse. In the normal state we must find absolute symmetry in the prominences and depressions. The eye then takes in the gluteal fold, which must not deflect to one or the other side; the supra-trochanteric dimples or depressions which vary in depth and area according to the leanness or obesity of the subject, preserving, however, in any instance a symmetrical appearance; the glutea-femoral folds, marked by fissures or creases indicating the junction posteriorly of the thigh with the trunk. These creases vary, too, according to the muscular or adipose development of the individual. As a rule the fissure is a bifurcated one, the upper curvilinear being the longer and extending from the perineum to the junction of the posterior with the anterior surface of the thigh, while the lower, nearly straight being shorter by one-half and leaving the upper about an inch from its femoral extremity, to extend an inch or two diagonally down the posterior aspect of the thigh. . . . We remember, too, that the law of symmetry must be recognized even in the fissures. Indeed, one can but admire the symmetrical arrangement of the lines and prominences so exquisitely drawn by nature in a pair of hips free from disease or deformity.

"One must not rest content with studying the parts already mentioned, but the eye will take in at a comparative glance the position of the trochanteric-prominence, the sacral region, the ilio-costal spaces and their relationship to the crista ilii, the size of the thighs in the upper one-third, and indeed, all the regions immediately connected with the hip. Soon one learns to observe all this at a glance and easily detects any departure, however slight, from the law of symmetry."

PATHOLOGY.

Hip disease begins most frequently as an ostitis and originates rarely as a synovitis. It has, however, from time to time been considered by various writers to originate in the synovial membrane, ligamentum teres and other ligaments, and even at the present day teachers are not fully agreed as to the original focus, but we believe the weight of opinion is in favor of a bone origin in children, while probably a synovitic is more common in adults. Gibney, Gross, Marsh, Barwell, Rust, Bryant, König, Volkmann, Annandale, hold the osseous origin; Sayre and Bilroth favor the synovial; while Owen, Holmes and others claim the ligamentous. Müller, as reported by König, analyzed specimens of sixty-one hip excisions and found in forty-seven cases osseous origin, three synovial, while in eleven he could not state the origin. Gibney reported an early autopsy in a case of double hip disease. In one joint the focus was in the head of the femur, while the other was in the acetabulum.

The process which takes place is a degenerative ostitis caused by the tubercle-bacilli; there is also a greater or less amount of formative activity, but the distinct pathological process is a degeneration.

The common form of tubercular infection of the epiphysis is the one spoken of as focal or encysted, when the first change is the formation of single or multiple foci of tubercular degeneration.

On section of the diseased epiphysis "the first noticeable change consists of a local hyperæmia of some part of the spongy tissue. There then appears in this hyperæmic area a small grayish translucent spot almost as small as one can see, which grows more and more gray and increases in size, while a zone of hyperæmic tissue develops around it, and the neighboring bone looks boggy from an excess of the transuded fluid. There is no synovitis; it is purely a localized ostitis.

"As the diseased focus grows larger it looks more yellow in spots, and shows at its center a tendency to cheesy degeneration and later in the history of the affection one finds nodules, varying in size from that of a pea to a hazlenut, which are filled with a putty like substance such as the cheesy material found elsewhere in the body, except that it contains spicules of bone from the trabeculæ, and in the larger *foci* pieces of dead bone of considerable size are found.

"Later in the history of the affection the tuberculous nodule breaks down into pus, and it is said that at this stage absorption has occurred, leaving nothing but a cavity filled with limpid serum." (Bradford and Lovett).

The absorption of the diseased focus is possible up to a late stage of the process, unless a sequestrum of bone of some size shall have formed; the pus becoming cheesy and calcified or possibly absorbed. Probably the most common course is for this cheesy mass to break down and discharge into the joint cavity or it may not enter the joint but break through the periosteum and discharge into the peri-articular tissues, when an abscess is formed, which, if not disturbed, will evacute spontaneously. The disease may end with this termination, granulation and cicatrization taking place, or it may continue indefinitely, first better then worse. The writer has seen several cases in which this condition had lasted from eight to twelve years, the disease apparently being arrested for months when some imprudence would bring on an exacerbation, which may continue for from a few weeks to as many months, this followed by a remission, then another exacerbation and so on. That this condition is less apt to follow joint affections, accompanied with abscess, than those without, statistics which are now being prepared on five hundred cases of "tumor albus" extending over a period of twenty years, will show.

It is abundantly proved by the microscope revealing the bacilli, in nearly all of the specimens examined for this purpose, that the disease is due to the tubercular bacilli. They are generally found in the "giant cells." They may be few in number and hence difficult to find.

In an abscess in a case at Volkmann's Clinic, twenty sections were examined and only two bacilli found. A number of orthopedists regard every case of hip disease as tubercular, although they are difficult to detect and exist in greater number in the early or stage of invasion than during the later stage when most examinations are made.

The inoculation of tissue from a scrofulous joint or that from a tubercular lung into a healthy animal, being followed by the same general and local condition as that produced by the inoculation of the pure culture of the bacilli, renders conclusive to the writer's mind the identification of scrofula or struma and tuberculosis.

ETIOLOGY.

That there exists in most, if not all, cases of joint disease an hereditary predisposition the writer is pretty firmly convinced. Figures which attempt to show what proportion of children inherit this tendency are unreliable, for the class of hospital patients from whom these statistics come are loath to acknowledge any inherited diathesis. The writer believes firmly in the identification of scrofula and tuberculosis.

"Gibney on the Hip," page 204, describes scrofula or struma as follows: "Struma, then, is a diathesis in an individual, either hereditary or acquired, and which renders its subject, especially in children, peculiarly vulnerable in certain tissues, viz.: the mucous membrane, the skin, the lymphatic system and the bones, and the inflammation which is so easily induced in the tissues named is remarkable for its great pertinacity and for products which are notably cellular in character, which present certain peculiar properties when inoculated in animals, and which instead of terminating in resolution or suppuration, extend locally and infect adjacent parts, developing either into tubercles or degenerating into caseation. "Call this diathesis a tendency if you will; it can scarcely be called a disease." The following quoted from Bradford and Lovett, proves that tuberculosis can be transmitted from parent to offspring:

"Landouzy and Martin, taking a six and one-half months' fœtus born of a phthisical mother, found it to all appearances perfectly free from tuberculosis; yet a piece of its lung put into a guinea pig's stomach caused general tuberculosis in four months, and inoculation was then carried through five animals. The cardiac blood from another fœtus caused the same tuberculosis in other guinea pigs. Again, one of these tuberculous guinea pigs gave birth to a litter, and a young one two days old was killed and appeared perfectly healthy; yet, pieces of its viscera inoculated into other guinea pigs caused general tuberculosis. And finally, the semen of a guinea pig thus rendered tuberculous was removed from the vesiculæ seminales with much care, and being inoculated into other guinea pigs caused tuberculosis."

Gibney analyzed 596 cases of joint diseases and found tuberculosis in one or both parents in 60 per cent.; an "acquired diathesis" in 30 per cent. He could only find one case which did not present either an inherited or acquired diathesis.

From the Alexander hospital of reports of 401 cases of hip disease, 24 per cent. had phthisis in the family history, and 35 per cent. were classed as traumatic.

Dr. C. F. Taylor, of New York, found in 845 cases of Pott's disease, 34 per cent. with a tubercular or scrofulous disease in the parents, and in 66 per cent. the disease came on in patients of a sickly diathesis. It has been proved that trauma to a joint of a tuberculous animal will cause tubercular joint disease where no such effect will follow in a healthy animal. This leads to the conclusion that traumatism applied to a strumous subject may be followed by tubercular joint disease, when in a healthy individual it will produce no such effect.

"Gibney observed 845 cases of spinal paralysis (a class of children subject to constant falls and injuries) for several years, and found only four complicated with joint troubles."

"Roser observed 100 children at Marburg with fracture of the elbow and in no case did tubercular disease follow."

The exanthemata are very fertile in issuing in a tuberculous joint disease. Measles and scarlatina being the most common, while diphtheria and pertussis contribute their share, especially the latter.

We may safely say that it is distinctly a disease of childhood, however; it is very rare under one year and probably never congenital. In 1880, Gibney reported 560 cases of hip disease. In 352 the disease began before the fifth year; in 290 between the third and fifth; thirty-nine after the tenth year, and only five after the thirtieth year; three at the fourteenth, and one each at the fifteenth and seventeenth years. (B. and L.)

Sex seems to play no part in the development. In 1,888 cases analyzed by Gibney, 909 were males and 909 females.

Dr. L. Emmet Holt collected 2,307 cases at the hospital for ruptured and crippled, and found 1,178 males and 1,129 females. The writer, during the past summer analyzed 470 cases of "tumor albus," and found 211 females; 259 males. Of the same 470 cases, 225 involved the right knee, 222 the left, while in twenty-three the side could not be ascertained.

Dr. Gibney, in closing the article, "Etiology in Diseases of the Hip," gives the following conclusions:

"1. A strumous diathesis, either hereditary or acquired, is the great predisposing cause of all chronic inflammatory bone lesions of the hip.

"2. That the disease may be excited by a fall or strain or wrench, exposure to cold, or by acute disease, an exanthem, for instance; with a prolonged convalescence.

"3. That in many cases no exciting cause can be found. . .

"The successful treatment of these maladies, attended by so much suffering, productive of so much deformity, much of which is often irremediable, and the mortaility, . . which is between 10 and 12 per cent., the successful treatment, I say, is the prize to the attainment of which all our labors should tend.

"That many diseases, essentially constitutional, demand local treatment, no sane man will deny; and with a proper understanding of the constitutional vice on which the local lesion depends for its existence, no sane man will assert that local treatment will meet all the indications."

SYMPTOMS.

It has been customary with a number of authors to divide hip disease into three stages, namely: First stage or period of invasion, extending between the initial lameness and the establishment of deformity ; the second stage, clinically speaking, extends from the establishment of permanent deformity due to muscular contraction to the deformity depending upon bony changes and displacements ; the third stage, that of real shortening, due mainly to the pathalogical changes in the bone, "the limb assuming positions consonant with the positions of the head, neck or acetabulum destroyed."—(Gibney.)

As it is not necessary to proper treatment, and as there is very little argument among writers as to what symptoms belong to each stage, it has not been deemed advisable to follow any such division.

The beginning of hip disease is generally most insidious. At first there may be only the very slightest amount of limping or stiffness of the limb on arising in the morning or after sitting for some time. In several cases the writer can call to mind, this slight limp would last a few days or weeks, then the limping would cease only to begin again in probably a week or month. This state of affairs may continue for months before medical advice is consulted. The parents consoling themselves with the thought, the child was suffering from severe "growing pains." Again, judging from histories given by parents, the disease may begin very abruptly, for I have frequently seen hips absolutely locked by muscular contraction in children who, the mother said, had been perfectly well the week previous ; but in dispensary hospital patients we are constantly reminded by their own statements that there is nothing so *unreliable* as absolute facts in regard to histories. We will admit that the disease may be more rapid of development in one than another, still we can hardly believe that a hip held firmly by muscular contraction, and probably fluctuation detected, can be the result of a week or month's disease, it matters not how severe the traumatism which may have started the pathological process.

The average case of hip disease runs about as follows : The slight halt, which is at first present in the morning, after a greater or less period, becomes a decided limp which is not overcome by exercise. The child, in standing or walking, supports itself principally on the sound limb with the offending one flexed at the hip and knee, with the ball and toes touching the floor, the flexion at the hip and knee acting as a spring to break the weight from the articular surfaces of the hip joint. At this stage pain may or may not be present.

During the early stage the child, wben apparently sleeping comfortably, may utter a moan, or a scream, and on going to him you will probably find him sleeping as calmly as possible. Children are troubled with "night cries" generally in the fore-part of the night, although they may occur at any time. They are caused from irritation reflex to the inflammation of the joint. One scream or six or eight may be given in succession, these, in a few moments followed by others, or no more may occur during the night. Some authors state as the average number of screams during the night one to twelve or fourteen, but I can call to mind two or three in whom their "night cries" averaged from ten to thirty-odd. This is a very important point in diagnosis. Generally the "night cries" come a little later than lameness and pain. Pain is a very uncertain symptom. Some cases are excessively painful, so much so that the child will scream, as though he were being murdered, on the slightest manipulation of the joint; while the next case may run its entire course without causing any annoyance from pain.

Muscular fixation is a very early accompaniment, due to the *reflex action* on the muscles controlling the joint. Upon this the most dependence in diagnosis is to be placed. All the motions of the thigh are either absolutely fixed or restricted; some motions may be more limited than others, for instance extension and abduction may be very slight, while flexion remains quite free.

Too much importance cannot be put to this muscular fixation, for in it we have an absolute key, we may say, to the progress of the disease, and also to it is due the malposition assumed, and from pressure of the head against the acetabulum, the wearing away of that bone and the head of the femur; hence we can readily see how this muscular rigidity is responsible for the awkward bony malpositions assumed in a bad, neglected case. The muscles becoming contracted and rigid crowd the head of the femur against the wall of the acetabulum. This constant pressure causes a "wearing away" of the articular surfaces, and further allows union to take place between the denuded and impacted surfaces; the patient recovering with an ankylosed limb in whatever position the head may have been held. The malpositions are flexion, abduction, adduction and rotation. While neither abduction nor adduction are characteristic of any particular stage, abduction is probably more common early in the disease, adduction later; though many cases begin and end with adduction. The limb may and generally does assume a combination of two or more of these; for instance flexion may occur combined with abduction and rotation or adduction and rotation. When ankylosis takes place in a case of double hip disease with even very slight malposition, progression is necessarily very awkward and labored. When both limbs are adducted and ankylosis has occurred, "cross-leg" progression, from inability to separate the limbs, renders one almost entirely incapacitated for locomotion. In assuming the erect position, abduction and adduction cause tilting of the pelvis, producing characteristic postures more easily recognized than described; while flexion causes lordosis in the lumbar spine in standing with the legs parallel; by resting on the well limb, or lying on the back with the diseased limb flexed, the lordosis may be overcome.

*Shortening*, an almost invariable accompaniment of hip disease, is largely due to *muscular contraction*. The pressure upon the diseased head conduces to a destruction of the articular surface and an alteration in the shape of the head and neck, allowing the trochanter to ascend above "Nelaton's line." When the acetabulum becomes diseased, a curious enlargement is apt to take place. The irritated muscles crowd the head against the upper border of the acetabulum, which produces an absorption of that portion of the rim resulting in an actual enlargement of the "acetabulum cavity" from below upwards, and the femur following, a pseudo-luxation is the result. Besides the shortening produced by destruction of bone in the femur and acetabulum, there is a decided trophic disturbance which results in retarding the bony growth and, at the same time, probably produces a certain amount of bone atrophy in extreme cases.

The shortening of the affected limb exists principally in the femur, although the tibia may share in it to a less extent also. The shortening of the thigh is generally about two-thirds of the whole, it may be less or again represent the whole amount.

Shaffer and Lovett claim, from an analysis of twenty-five cases of cured hip-disease, that the difference in the length of the legs almost always increases slightly after the disease is cured.

This leads us to another and *very early* and decided symptom of hip-disease, namely, muscular atrophy. This is claimed by some to be reflex to the diseased joint, by others to disuse. As this presents itself at such an early stage of the disease and before immobilization or rest of the affected limb could possibly have any effect in producing the amount of atrophy existing, it appears to the writer nothing more than rational that we should look to some nervous origin for this condition; to some trophic change produced by a reflex from the diseased joint.

In support of this, "if the muscles of the thigh are tested for contractility to the irritation to the faradic current it will be found that the contractility is markedly diminished."—(Bradford and Lovett.)

Atrophy of the muscles is an invariable accompaniment of a diseased joint and may begin quite early and proceed very rapidly, or it may be absent for weeks or months; though in my fourteen months' experience at the hospital for Ruptured and Crippled, New York, I cannot recall a case in which there was no atrophy, even on the first examination.

The writer realizes the fact that in most dispensary cases the disease may have existed for weeks or months before surgical advice is sought; while in private practice one has far better opportunity for seeing these cases at their invasion.

The inguinal glands frequently become enlarged, and occasionally to such an extent as to impede the venous return. They are occasionally the seat of superficial abscess. In very severe cases the tissues in the hip, the peri-articular tissues, become

œdematous, this may disappear or result in the formation of an abscess.

In a large proportion of cases suppuration takes place and is frequently accompanied by very severe pain. Abscesses may be peri-articular or articular according as the initial focus of the epiphysis extends outside of or into the joint, or an abscess, originally peri-articular, may later involve the joint. The formation of an abscess is frequently without constitutional symptoms although it may be accompanied by slight fever. After the pus has left the joint cavity, it burrows through the thigh muscles to reach the surface, where it appears as a fluctuating tumor of varying size. After having reached the skin, the same becomes red, glazed, thin and ulcerates at one or more points, allowing spontaneous evacuation. The abscess may terminate with its evacuation, but far more frequently continue to discharge from a fistulous opening for months or years. The pus may dissect through the tissues and point at the anterior border of the tensor vaginæ femoris, or back of the great trochanter or lower border of the gluteus maximus, or on the inside of the thigh in front of the adductor tendons; it may descend and open in the popliteal space or ascend in the sheath of the psoas and discharge above Poupart's ligament, or it may discharge into the rectum. Improper treatment, malposition, firm muscular fixation and a painful condition of the joint conduce, it has been claimed, to the formation of abscess.

Out of three to four hundred cases of hip disease treated by ambulatory methods at the Out Door Department of the Children's Hospital, Boston, only thirteen reported as having developed abscess. (B. and L.) There, as soon as malposition becomes marked, the patient is admitted to the hopital, and an attempt is made by rest and gradual extension to overcome the deformity. When this has been accomplished the patient is again allowed to get up and go about as usual with splint adjusted.

The general condition may remain good throughout the entire course of the disease, or the child being in apparently robust health at the beginning, after a few months becomes pale and the appetite capricious, anorexia following, the patient losing flesh and strength; at this stage it may stop; the appetite being regained, improvement in the general condition following; "but at other times decided constitutional disturbance" may supervene.

Very few cases of hip disease run a smooth even course; *i. e.*, they will for some time run without an untoward symptom, and then without any apparent cause an exacerbation will occur, soon followed by a remission and so on; or the case may be characterized by its extreme severity, when as suddenly a remission in these severe symptoms will occur, only to be followed soon with probably increased severity.

DIAGNOSIS.

The diagnosis of hip disease rests upon the following symptoms: 1, stiffness; 2, lameness; 3, atrophy; 4, pain; 5, malpositions; 6, swelling. The chief diagnostic sign in hip disease is muscular rigidity, "*the presence of stiffness of the joint or limitation of its proper arc of motion*, when the limb is passively manipulated." There can be no disease, except probably in its very incipiency, without limitation of motion. This is not due to adhesions, but to a tonic spasm of the muscles controlling the joint, and disappears under an anæsthetic.

At times great care and skill are required to detect slight limitation of motion, *especially* in *young children*, who are apt to become frightened and resist any attempt at examination. When due to fright, however, resistance is made to all motions of the limb, and to both limbs alike; should this be overcome, or should the child's attention be withdrawn from its limb, then resistance to rotation of a flexed thigh and to the *extremes* of the normal motions *will not* be encountered; whereas, in hip disease it is at the *extremes* of the arc of normal movement that *resistance* is *experienced.* Valuable information can be obtained from a comparison of the movements of the two limbs.

Flexion may be determined by flexing the sound limb upon the abdomen to its utmost limit, then repeating this with the suspected limb, keeping the sound limb firmly on the table. By repeating the flexion of the sound limb and extending the other until the pelvis begins to move, the degree of extension may be obtained. Extension may also be determined by placing the two limbs parallel so that the popliteal space of both will rest against the table; normally there will be no change in the back, but should there be contraction of the psoas and iliacus, the back will be arched as the diseased limb is pressed down. Extension and hyper-extension may be determined by examining the patient lying upon the belly. With one hand placed firmly upon the sacrum, with the other alternately raising the thighs from the table, any difference in the amount of extension can readily be detected.

Abduction and adduction can be determined, the patient lying upon his back, by firmly grasping the anterior superior spine of the ilium, on the sound side, with one hand, and with the other adduct and abduct to the extreme limit of the suspected limb; this should then be repeated on the sound limb. In the early stages limitation to rotation is not so easily detected. It is determined though as follows: Flex the thigh on the abdomen, then rotate the femur and abduct. This will frequently show a limitation as compared with the sound side.

Limp is another early symptom of considerable value. In fact, hip disease cannot exist without giving rise to it, but, as stated above, in the early stage it may be intermittent, or barely perceptible.

It is worse in the morning than at night. Muscular atrophy occurs very early. Its absence, however, does not necessarily exclude hip disease. The obliteration of the folds and creases of the haunch, and the flabby and sunken appearance of the buttock, are largely due to this atrophy.

Atrophy may be determined by inspection, if it be decided, or by palpation, the muscles giving a soft and flabby feel, or with the tape. Accuracy is always preferable, hence the circumference of both limbs is taken at corresponding distances from some bony prominence, as the anterior superior spine of the ilium, the patella or malleolus. The circumference of thigh, knee and calf should be taken. The thigh will give from one-fourth of an inch to an inch of difference, the knee very little if any, while the calf will be about half that of the thigh.

The location of pain, as well as its intensity, is variable, but it is most frequently referred to the inside and front of the thigh, near the knee, or to the knee joint solely.

The intimate anastomoses of the sciatic obturator and anterior crural nerves are thought by many to offer the best explanation of this. Occasionally the pain extends into the leg, following the course of the sciatic nerve. Pain referred to the hip is indicative of suppuration, or due to nervous hyperæsthesia. Tenderness on percussion of the joint is neither an early or constant symptom. Percussion, as an aid to diagnosis, is an unjustifiable procedure, as the information gained will not begin to compensate for the damage done in jamming the head of the femur against the acetabulum.

Swelling is not very common in the early stage unless the disease has been unusually severe and rapid. In the later stages œdema may become quite a prominent feature. The inguinal glands may become enlarged and suppurate on one or both sides.

The abnormal positions, due to muscular contraction and bony ankylosis, are abduction, adduction, flexion and rotation, or there may be two or more combined. Abduction or adduction may exist to a marked degree and still not be recognized by the patients as such, they attributing the difficulty of locomotion to lengthening or shortening of the diseased limb. The trouble really lies in the tilting of the pelvis, which causes the limb to appear shorter if adducted, longer when abducted. This is termed the practical shortening or lengthening and may be measured by taking the distance from the umbilicus to the malleoli. The tilting of the pelvis may be recognized by mere inspection or by placing a tape from one ant. sup. spine of the ilium to the other, which should be at right angles to a second line or tape placed from the umbilicus to the symphysis pubis. Real or bone shortening results from retarded growth, or from the destruction of bone in the joints. The amount of destruction which has been done to the head of the femur and acetabulum may be estimated by determining the amount of subluxation. The patient lying upon the well side with the affected thigh slightly flexed, "Nelaton's line" is drawn over the diseased hip, *i. e.*, a line drawn from the ant. sup. spine to the most prominent part of the tuberosity of the ischium should pass just above the upper margin of the trochanter major. Should this be above the line it is evidence of so much subluxation.

The real shortening or lengthening is determined by taking the distance from the ant. sup. spine to the corresponding malleolus. The amount of abduction and adduction was formerly and is still to some extent taken by means of the goniometer; this not being always at hand and frequently inaccurate, a method has been devised by which it is possible to estimate the angle of deformity with the tape measure and a table of figures.

The real and apparent lengths of both limbs are taken. The difference between the differences in the two kinds of shortening is noted. The only other measurement required is that between the ant. sup. *spine*. This obtained, turning to the table, we follow the line which represents the difference in inches between the apparent and real shortening, until it intersects the line, giving the pelvic breadth, when we have the degree of either abduction or adduction.

"*If the practical shortening is greater than the real shortening, the diseased leg is adducted; if less than real shortening, it is abducted.*"

For example, the real length of the right limb being 26 inches, left limb 25, apparent length of right limb 28½, left limb 26 inches; difference in real shortening 1 inch; apparent shortening 2½ inches, hence the difference between the differences is 1½ inches; pelvic breadth, 8 inches. Now, if we follow the line for 1½ inches, until it intersects the pelvic measurement of 8 inches, we find 11, which means 11° of abduction of the diseased thigh.

DISTANCE BETWEEN ANT. SUP. SPINE IN INCHES.

| Difference in inches between real and apparent shortening. | 3 Deg. | 3½ Deg. | 4 Deg. | 4½ Deg. | 5 Deg. | 5½ Deg. | 6 Deg. | 6½ Deg. | 7 Deg. | 7½ Deg. | 8 Deg. | 8½ Deg. | 9 Deg. | 9½ Deg. | 10 Deg. | 11 Deg. | 12 Deg. | 13 Deg. |
|---|---|---|---|---|---|---|---|---|---|---|---|---|---|---|---|---|---|---|
| ¼ | 5 | 4 | 4 | 3 | 3 | 2 | 2 | 2 | 2 | 2 | 2 | 2 | 2 | 1 | 1 | 1 | 1 | 1 |
| ½ | 10 | 8 | 7 | 6 | 5 | 5 | 4 | 4 | 4 | 4 | 4 | 4 | 4 | 3 | 3 | 3 | 3 | 2 |
| ¾ | 14 | 12 | 11 | 10 | 8 | 8 | 7 | 7 | 6 | 6 | 5 | 5 | 5 | 4 | 4 | 4 | 3 | 3 |
| 1 | 19 | 17 | 14 | 13 | 11 | 10 | 9 | 9 | 8 | 7 | 7 | 7 | 6 | 6 | 6 | 5 | 5 | 4 |
| 1¼ | 25 | 21 | 18 | 16 | 14 | 13 | 12 | 11 | 10 | 9 | 9 | 8 | 8 | 7 | 7 | 7 | 6 | 6 |
| 1½ | 30 | 25 | 22 | 19 | 17 | 15 | 14 | 13 | 12 | 12 | 11 | 10 | 10 | 9 | 9 | 8 | 7 | 7 |
| 1¾ | 36 | 30 | 26 | 23 | 20 | 18 | 17 | 15 | 14 | 13 | 13 | 12 | 11 | 10 | 10 | 9 | 8 | 8 |
| 2 | 42 | 35 | 30 | 26 | 23 | 21 | 19 | 18 | 16 | 15 | 14 | 14 | 13 | 12 | 12 | 10 | 10 | 9 |
| 2¼ | | 40 | 34 | 30 | 26 | 24 | 21 | 20 | 19 | 17 | 16 | 15 | 14 | 14 | 13 | 12 | 11 | 10 |
| 2½ | | | 39 | 34 | 29 | 27 | 24 | 22 | 21 | 19 | 18 | 17 | 16 | 15 | 14 | 13 | 12 | 11 |
| 2¾ | | | | 38 | 32 | 29 | 27 | 25 | 23 | 21 | 20 | 19 | 18 | 17 | 16 | 14 | 13 | 12 |
| 3 | | | | 42 | 35 | 32 | 29 | 27 | 25 | 23 | 22 | 21 | 19 | 18 | 18 | 16 | 14 | 13 |
| 3¼ | | | | | 39 | 36 | 32 | 30 | 27 | 26 | 25 | 22 | 21 | 20 | 19 | 17 | 15 | 14 |
| 3½ | | | | | | 40 | 35 | 33 | 30 | 28 | 26 | 24 | 23 | 22 | 21 | 19 | 17 | 16 |
| 3¾ | | | | | | | 38 | 35 | 32 | 30 | 28 | 26 | 25 | 23 | 22 | 20 | 18 | 17 |
| 4 | | | | | | | 42 | 38 | 35 | 32 | 30 | 28 | 26 | 25 | 23 | 21 | 19 | 18 |

A somewhat similar method may be used for measuring the degree of flexion. "The patient lies upon a table, flat upon his back, and the surgeon flexes the diseased leg, raising it by the foot until the lumbar vertebræ touch the table, showing that the pelvis is in the right position. The leg is then held for a minute at that angle, the knee being extended, while the surgeon measures off two feet on the outside of the leg with a tape measure, one end of which is held on the table so that the tape measure follows the line of the leg. From this point, on the leg, where the two feet reaches by the tape measure, one measures perpendicularly to the table and the number of inches (in the perpendicular line) can be read as degrees of flexion of the thigh by consulting the following table. For instance, if the distance between the point on the leg and the table is 12½ inches, it represents 31° of flexion deformity of the thigh."

| Inches. | Degrees. | Inches. | Degrees. | Inches. | Degrees. | Inches. | Degrees. |
|---|---|---|---|---|---|---|---|
| 0.5 | 1 | 6.5 | 16 | 12.5 | 31 | 18.5 | 51 |
| 1.0 | 2 | 7.0 | 17 | 13.0 | 33 | 19.0 | 52 |
| 1.5 | 3 | 7.5 | 19 | 13.5 | 34 | 19.5 | 54 |
| 2.0 | 4 | 8.0 | 20 | 14.0 | 36 | 20.0 | 56 |
| 2.5 | 6 | 8.5 | 21 | 14.5 | 37 | 20.5 | 58 |
| 3.0 | 7 | 9.0 | 22 | 15.0 | 39 | 21.0 | 60 |
| 3.5 | 9 | 9.5 | 24 | 15.5 | 40 | 21.5 | 63 |
| 4.0 | 10 | 10.0 | 25 | 16.0 | 42 | 22.0 | 67 |
| 4.5 | 11 | 10.5 | 27 | 16.5 | 43 | 22.5 | 70 |
| 5.0 | 12 | 11.0 | 28 | 17.5 | 45 | 23.0 | 75 |
| 5.5 | 14 | 11.5 | 29 | 17.5 | 47 | 23.5 | 80 |
| 6.0 | 15 | 12.0 | 39 | 18.0 | 48 | 24.0 | 90 |

The writer has had no personal experience with this method of determining flexion, having depended entirely upon Thomas' tests and the goniometer, although he is satisfied that at times it may prove very valuable.

The patient lying upon the back, the sound limb is flexed on the abdomen and held firmly; at the same time the diseased limb is extended to the extreme limit, care being taken to keep the lumbar spine in contact with the table. In this position one arm of the goniometer is placed along the axis of the thigh, the other on the side of the body, so that the upper end points to the center of the axilla. The degree of flexion is then read off the scale, which, in this position, will be over the trochanter major.

(*Concluded next issue.*)

